# The Eyes and the Teeth; Have They Any Nervous Connection?

**Published:** 1876-05

**Authors:** J. E. Cravens


### ARTICLE VI.

### *The Eyes and the Teeth; have they any Nervous Connection?*

BY J. E. CRAVENS, D. D. S.

In the March number of the *Missouri Dental Journal*, Dr. J. Campbell, of St. Louis, reportee a very interesting and highly instructive case of "Temporary loss of sight, caused by pulpits of a canine tooth," the patient a lady about thirty-five years of age and moderately good health. Dr. Campbell requested comments on the case reported. Dr. Campbell's reported treatment was to remove the tooth, the sight returning again in the lapse of a few months this however, was several years since. I do not believe the Doctor would extract the tooth in a similar case to day, although he does not say. In this connection I deem it pertinent to report a somewhat analogous case of a recent date, in my own practice.

About the middle of November, 1873, Dr. S., physician of this city, escorted a lady to my office for the purpose of placing her under my charge for the treatment of her teeth, hoping thereby to benefit her eyes. It seems that she was utterly unable to distinguish anything in daylight, and had been shut up in a dark room during the daytime for several months. She was about twenty-five years old, never had been troubled by her eyes previous to the instance referred to. The history I obtained from her of the case from incipiency was as follows: She resided in Wyandotte, Kansas, suburban to Kansas City. The right superior lateral incisor became troublesome from hyperæsthesia in cavity in palatal surface; after applying creosote until her mouth was quite sore and the pain in the tooth only aggravated, she called the family physician who advised her to consult (a sort of drunken itinerant,) the only dentist in the place. This dentist, she said, put something into the cavity in palatal surface to destroy the nerve. She could not say what he used, except she detected the odor of creosote. Troubles however, began to thicken about the case thenceforth; the tooth became so sensitive to thermal changes, especially depressed temperature, that she found it necessary to cover the labial surface of that individual tooth with beeswax. Contact with air in speaking was distracting. Both eyes become sensitive to the sun's rays, requiring constant protection by a green veil when out doors or near an unshaded window.

Gradually this sensitiveness of the tooth increased, that of the eyes still more rapidly, until the vision was affected, resulting in total blindness in daylight. She could see a little by clear moon or starlight or soft artificial light, but she experienced immediate suspension of sight and suffered acute pain if by chance the rays fell upon her eyes direct from the illuminating object. When her eyes first became affected her physician concluded to remove all carious teeth from her mouth, but abandoned the project after extracting three or four teeth from the left side above, and not affording any relief. He then referred her to Dr. S., of this city, who brought her to my office. Dr. S., suggested the possibility of a nervous connection of an indirect character, but exceedingly sympathetic, between the incisor in question and the eye. He could detect nothing abnormal in the appearance of any part of the eyes, which were of that deep lustrous black that a little distance gives the impression of an absence of any such distinction as pupil and iris; neither were there any indications of conjunctivitis, so much for the eyes. The diagnosis of the incisor discovered two cavities, one already mentioned in palatal surface, the other in mesial proximation, separated by spicula of enamel, of half a line width, but connected by a small *sous*-enamel passage; both cavities were quite shallow, having perhaps, a dentinal depth of half a line. I found nothing like sensitive dentine in manipulating the excavator in the cavities, but cold air or water caused a paroxysm of pain, apparently in the tooth. In color the tooth was not different from the neighboring central and canine, both of which were perfect and unaffected by the troubles of the lateral; concentrated sun rays showed the same translucence as the healthy neighbors. The tooth was apparently alive—apparently dead. To solve the problem I drilled through the palatal cavity into the pulp chamber. No pulp was there. The chamber was filled with a slightly yellow fluid of the consistency of glycerine, with an exaggerated odor of fresh egg. The fluid occupied the canal of the root. At the apex of the root I found the sensitive condition peculiar to that point just after the removal of the pulp tissue therefrom. I dressed the canal to the apex with a saturated solution of iodine in creosote—both officinal—stopped the pulp cavity with beeswax, and applied the same solution freely to the mucous membrane over the root. The lady's husband was present, to whom I gave a small vial of the solution referred to, and a broach nerve plugger and raw cotton, also imparting instructions as to the manner of changing the applications, directing that it be done daily for four or five days, at which time the patient should call again. The patient called after an absence of about two weeks, the 3d of December, 1873, when she reported the tooth perfectly comfortable. Her husband had continued changing the application for several days as directed, when the susceptibility to thermal changes ceased; the cavity was sealed with beeswax, the last application left in, to be removed by myself, as I had requested in order that I might detect any fetor if present.

At that sitting I filled the canal and both cavities of decay with gold. At time of introducing the fillings the lady had experienced less pain from the sun's rays, and no decided irritation from artificial light, although her vision was still impaired, but improved greatly.

August 31st, 1874, the patient called to have another tooth filled, and reported that the lateral incisor previously operated on had given no further trouble, and her eyes had improved so much that she needed no veil or protection of any sort except when the sunlight was intense, and then one thickness of ordinary green veil was ample. On September 5th, 1874, her husband called at my office to say that her eyes and tooth were apparently in as good condition as ever, bright sunshine having no other effect on her eyes than is usually experienced by people in general.—*Dental Register.*

---

